# Transition of a microRNA from Repressing to Activating Translation Depending on the Extent of Base Pairing with the Target

**DOI:** 10.1371/journal.pone.0055672

**Published:** 2013-02-06

**Authors:** Ashesh A. Saraiya, Wei Li, Ching C. Wang

**Affiliations:** Department of Pharmaceutical Chemistry, University of California San Francisco, San Francisco, California, United States of America; National Institute of Health, United States of America

## Abstract

MicroRNAs are major post-transcriptional regulators of gene expression. Here we show in the ancient protozoan Giardia lamblia a snoRNA-derived 26-nucleotide microRNA, miR3, which represses the translation of histone H2A mRNA containing an imperfect target but enhances translation when the target is made fully complementary. A stepwise mutational analysis of the fully complementary target showed that the activating effect of miR3 was significantly reduced when a single nucleotide at the 5′-end of the target was altered. The effect of miR3 became repressive when 12 of the nucleotides lost their complementation to miR3 with maximum repression reached when only 8 base-pairs remained between the miR3 seed sequence and the target. A synthetic 8-nucleotide RNA oligomer of the miR3 seed sequence was found capable of exerting a similar Argonaute-dependent translational repression. This is the first report showing a correlation between the extent of base-pairing with the target and a change in miRNA function.

## Introduction

MicroRNAs (miRNAs) are small, non-coding RNAs involved in post-transcriptional gene regulation. The action is mediated through base-pairings between an Argonaute protein-complexed miRNA and an imperfectly matched target site usually located at the 3′-end of a target mRNA [1], [2], resulting in translational repression followed by deadenylation and degradation of the mRNA [3], [4]. Approximately 1% of the genes in animals have been found to encode miRNAs, which are often highly conserved across a wide range of species [1].

Since the original discovery that miRNAs were essential regulators of development in Caenorhabditis elegans [5], there have been increasing numbers of examples indicating that miRNAs are involved in many divergent cellular processes. To cite but a few examples, they modulate hematopoietic lineage differentiation [6], regulate brain morphogenesis in zebrafish [7] and induce cell proliferation [8] and apoptosis in Drosophila [9]. Variations in miRNA expression levels are thus often associated with various types of tumors [10], [11]. Viruses often use miRNAs in controlling their host cells [12], whereas the host also use miRNAs to block essential viral functions [13]. miRNAs may thus constitute an important new layer of regulation of gene expression in many eukaryotes.

Rules for miRNA-target interactions are still unclear, but some general guidelines have been established. Base-pairings of the 5′ end of miRNA at positions 2–8, the seed sequence, with the target is usually crucial for miRNA targeting. The duplex thus formed has been postulated to function as a nucleation site for the remainder of the miRNA-target interaction, though experimental support for such hypothesis is still lacking [1], [14], [15]. The 3′- sequence downstream from the seed sequence of a miRNA, however, often forms imperfect base-pairings containing mismatches, GU wobbles and bulges with the target. These imperfections in complementation have been routinely identified among miRNA-target complexes, suggesting that perfect base-pairings are not necessary for miRNA function [1], [14], [15]. Comparative analysis of miRNAs in Drosophila [14] and mammals [16] indicated that the 3′- pairing of functional miRNAs to their targets are no more than expected by chance. Mammalian targets for miRNAs can be readily predicted around the 3′-untranslated regions (UTRs) of transcripts by searching for conserved matches to the 7-nucleotide (nt) seed sequences in miRNAs [16].

Though an imperfectly matched miRNA-mRNA pair with central bulges leads to translational inhibition followed by deadenylation and degradation of the mRNA [3], [4], a perfectly complementary pair tends to promote mRNA slicing by the Argonaute protein [17], [18]. There are, however, occasional exceptions to this rule [19], [20], which could be attributed to the presence of Argonaute proteins lacking endonucleolytic activity [21]. Loading of a miRNA onto such a protein could lead to only translational repression of the target mRNA.

Several functional miRNAs have been recently identified in Giardia lamblia, a deeply branched protozoan parasite responsible for the common diarrheal disease giardiasis [22], [23], [24], [25]. These miRNAs are derived from either snoRNAs or by a canonical biogenetic pathway mediated by the *Giardia* Dicer protein, GlDcr [22], [23], [24], [25]. The miRNAs range in size from 26 to 28 nts, which is in good agreement with the size estimated from the crystal structure of GlDcr [26]. There is a single Argonaute protein (GlAgo) in Giardia. It forms complexes with the miRNAs and mediates their actions on mRNAs carrying imperfectly complementary target sites by repressing mRNA translation without apparent degradation. Reporter mRNAs with fully complementary target sites to miR4 [24] and miR5 [22] were tested previously and were found to be translationally repressed without mRNA degradation, suggesting that GlAgo may lack endonucleolytic activity and the miRNA action in *Giardia* may be confined to translational repression regardless of the extent of base-pairings between the miRNAs and their targets (see Discussion).

Here we report in *Giardia* another snoRNA-derived miRNA, miR3, that has a 26 nt sequence and represses translation of a histone H2A mRNA carrying an imperfectly complementary target site by ∼20%. It has similar features to all the other miRNAs previously identified in Giardia [22], [23], [24], [25]. However, when we altered the target site sequence to fully complement miR3, the latter showed an unexpectedly strong activating effect on translation of the mRNA. A stepwise mutational analysis of the target sequence indicated that a full 26 nt complementation with miR3 is required for maximum activation, whereas base-pairing with just the 8-nt seed sequence of miR3 results in maximum repression. Both actions are dependent on the presence of GlAgo.

This is, by our knowledge, a novel observation, which may help us further understand the mechanism behind miRNA regulation of gene expression. The dramatic transition from repressing to activating translation with increased base-pairings between the miRNA and the target may provide a good opportunity to study the detailed mechanisms of miRNA-mediated regulation of mRNA translation.

## Results

### miR3 is a snoRNA-derived miRNA

miR3 was originally identified during the cloning of size fractionated small RNAs from *Giardia* trophozoites [25]. It has a length of 26 nts (5′ GCA GAC AAC GCA UCA CCG CUC UGA CC 3′), is derived from the 3′-end of a conserved orphan Box C/D snoRNA, GlsR16, and requires the GlDcr for processing into the mature form [25]. To complete the characterization of this small RNA, we used fluorescence *in situ* hybridization (FISH) to indicate that miR3 is primarily localized to the cytoplasm while GlsR16 is concentrated in the nucleolus-like organelles of *Giardia* trophozoites (Figure S1A). It confirms that GlsR16 is most likely a snoRNA localized to the nucleolus and that miR3 is a cytoplasmic product derived from GlsR16.

A 2xHA tagged *Giardia* Argonaute (HA-GlAgo) protein expressed in Giardia trophozoites was co-immunoprecipitated with a small RNA band of ∼26–30 nts [22], [24]. Using a 5′ nuclease qPCR assay (Taqman) (Figure S1B), the presence of miR3 in the GlAgo-associated small RNA band was demonstrated, suggesting that miR3 could be a functional miRNA (Figure S1B).

### Identification of the Putative Targets of miR3

**Figure 1:**
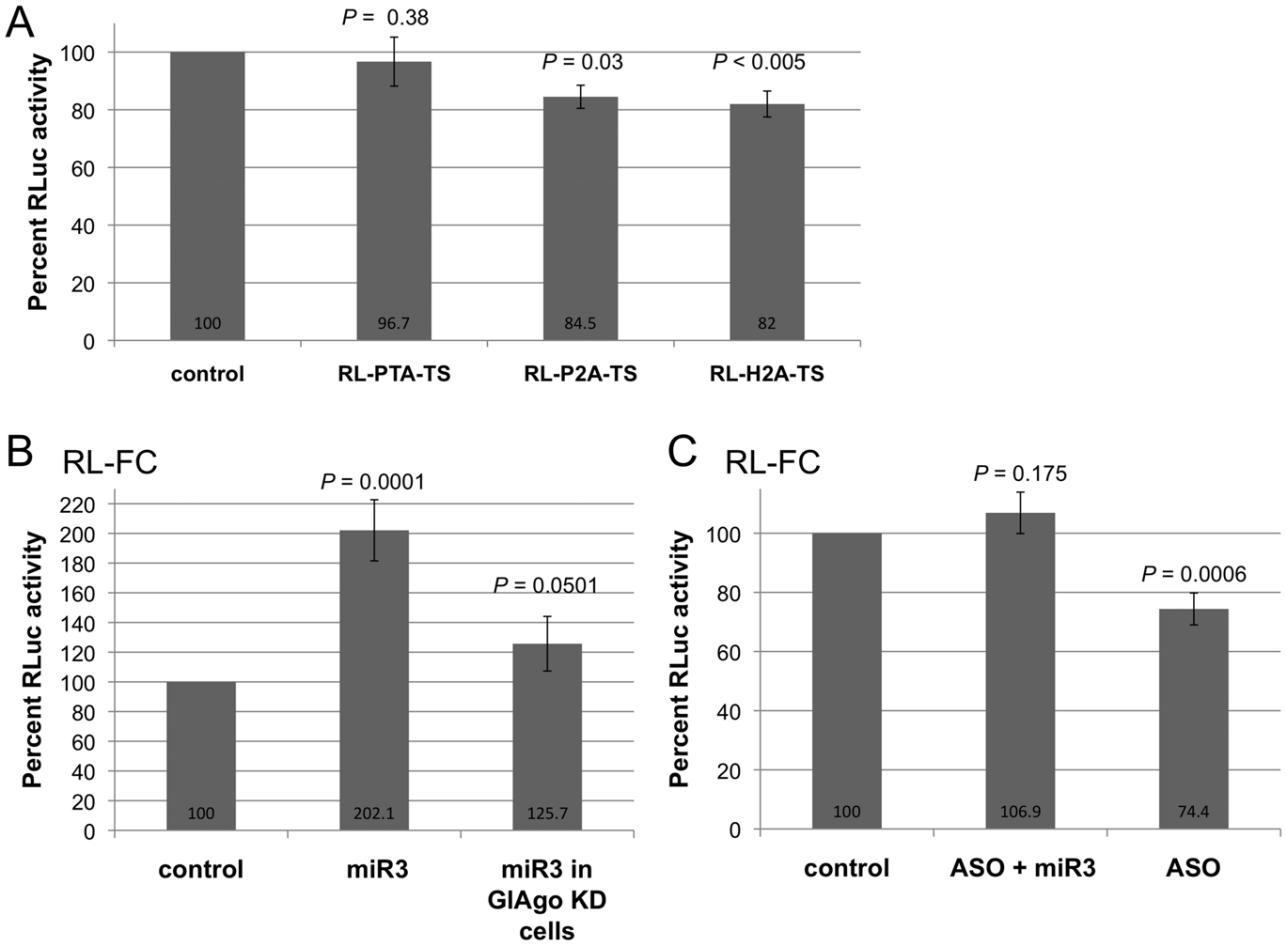
Testing the putative targets of miR3. **A)** Potential miR3 target sites identified by the miRanda program were tagged to the 3′ UTR of an RLuc transcript and tested against synthetic miR3 in transfected *Giardia* trophozoites. The three target sites were derived from the 3′-ends of potassium-transporting ATPase (PTA-TS), Ser/Thr phosphatase 2A (P2A-TS) and histone H2A (H2A-TS), respectively. The RLuc activity, reflecting efficiency of miR3 repression, suggests a reversed correlation with the predicted miRanda scores and free energy of binding of the targeting sites (see Table 1). The control represents the RLuc activities expressed by the chimeric transcripts without introducing miR3 into the cells. They were each set at 100% and presented in a single column as the “control”. **B)** The expression of RL-FC is activated by two-fold in the presence of miR3 in *Giardia* trophozoites. This activation is significantly reduced in the GlAgo KD cells. The control shows expression of RL-FC without miR3 and is set at 100%. **C)** Co-transfection of *Giardia* with an equivalent amount of miR3 2′ O-methyl antisense oligo (ASO) and miR3 abolishes the miR3-mediated activation of the RL-FC expression. Transfection with miR3 ASO alone causes a decrease in RLuc activity by ∼25%, suggesting that endogenous miR3 is also activating expression of RL-FC. The control indicates expression of RL-FC without ASO or miR3 and is set at 100%. These data represent the mean +/− SD from three independent experiments. The *P*-values were determined using an unpaired student t-test and compared to the control.

We used the miRanda (version 3) program to identify potential targets of miR3 in the Giardia genomic database [27]. Fifty nts upstream and 100 nts downstream of the stop codon from each open reading frame (ORF) in the *Giardia* database were chosen for the search [28]. The resulting hits were further filtered to remove targets with less than 5 complementary bases in the seed sequence while allowing one G:U wobble pair. Of the 4969 ORFs in the *Giardia* genome database (version 1.1), 127 genes were found to carry potential miR3 targets (Table S1), of which 55 encode annotated proteins while the remaining 72 encode for hypothetical proteins. Among the former, there were two variant-specific surface proteins (VSP), one high cysteine membrane protein and a spectrum of other proteins without any apparent correlation among their biological functions (Table S1). This is in contrast to the other previously characterized *Giardia* miRNAs, which act on large numbers of VSP transcripts [23], [24], [25]. No target with complete complementarity to miR3 was identified. We chose three putative targets of miR3 for further analysis based on their relatively high miRanda scores and low free energies of miR3 binding. All three target sites are positioned across the ORF and the 3′-UTR of the gene. These sites include a potassium-transporting ATPase alpha chain 1, a Ser/Thr phosphatase 2A 65kD regulatory subunit A and histone H2A (Table 1). Two copies of each of the potential target sites were inserted into the 3′ UTR of a *Renilla* luciferase (RLuc) reporter transcript, introduced into Giardia trophozoites together with chemically synthesized miR3 by electroporation, and assayed for RLuc expression (Figure 1A). The potassium-transporting ATPase target site (RL-PTA-TS) has the most extensive base pairings with miR3 (Table 1), but RLuc expression (∼3%) was not appreciably affected by miR3 (Figure 1A). Increased repression of RLuc activity (∼15%) was observed for the Ser/Thr phosphatase 2A (RL-P2A-TS) target site, whereas the histone H2A (RL-H2A-TS) target site with the lowest miRanda score (Table 1) showed the highest repression by miR3 (∼18%) (Figure 1A).

**Table 1:** Three putative targets of miR3 selected from the *Giardia* genomic database for testing.

MiRandascore	ΔG (kcal/mole)	Target site location(nts)^a^	Gene ID	Encodedprotein	Target site b
178	−33.52	38–63	GL50803_96670	Potassium-transportingATPase alpha chain 1	miR3: 3′ CCA GUCUCG CCA CUA CGCAAC AGA CG
					| **:** **:** ||| || ||| ||| ||| |
					5′ ACU GGU CUUGG**U** **AA**U GCGUUG UCU GG
177	−31.62	47–73	GL50803_7439	Ser/Thr phosphatase 2A,reg sub A	miR3: 3′ CCA GUC UCGCCA CUA C– GCAACA GAC G
					| **:**| ||| | ||| | ||| ||| |||
					5′ AG**U** **AG**G AGC UG- GAT GCC CGU UGU CUG C
134	−20.93	37–63	GL50803_14256	Histone H2A	miR3: 3′ CCA GU- CUC GCCACU ACG CA- ACA GAC G
					|| || | ||| |**:** || ||| |||
					5′ UCG CAG GAUCUU **UGA** –GU GUCUGU CUG U

a - The numbering is based on the 150 nts chosen for analysis (50 nts upstream and 100 nts downstream of the stop codon).

b - Target sites were identified with the miRanda program. The stop codon is shown in **bold**.

### Enhanced Expression of a Reporter Transcript Carrying a Fully Complementary Target Site for miR3

This somewhat unexpected data in Figure 1A appeared to suggest that the extent of sequence matching between miR3 and its target does not necessarily dictate the efficiency of miR3 inhibition of RLuc expression. To test this possibility, we incorporated two 26 nt target sites fully complementary (FC) to miR3 into the RLuc transcript 3′-UTR (RL-FC) and tested if miR3 would have any effect on its expression in Giardia. The results presented in Figure 1B indicate that, instead of an inhibitory effect, miR3 enhanced the expression of RL-FC by as much as two-fold. There was no significant change in the level of RL-FC transcript when co-transfected with miR3 (Figure S2B), suggesting that miR3 enhances the translation of the RL-FC transcript. The experiment was then repeated in a Giardia mutant cell line (GlAgo KD), in which the GlAgo protein was reduced by a giardiavirus-mediated antisense-hammerhead ribozyme knockdown of the GlAgo mRNA [25]. The experimental outcome showed that miR3 enhanced expression of RL-FC by only ∼26% in these cells (Figure 1B), indicating that the miR3-mediated enhancement of translation is dependent on GlAgo. To further verify whether the stimulatory effect of miR3 is through hybridizing to the fully complementary target, a 2′ O-methyl antisense oligo of miR3 (ASO) was co-introduced with miR3 into the Giardia cells. The ASOs are known to form stable duplexes with the corresponding miRNAs to block their functions [29]. The introduction of an equivalent amount of ASO with miR3 into Giardia resulted in virtually no effect on the expression of RL-FC (Figure 1C). When only the ASO was introduced, there was a ∼25% reduction in RL-FC expression, suggesting the presence of endogenous miR3 in Giardia is capable of enhancing the expression of RL-FC by 25% (Figure 1C).

#### Enhancement of histone H2A gene expression carrying a target site fully complementary to miR3

**Figure 2:**
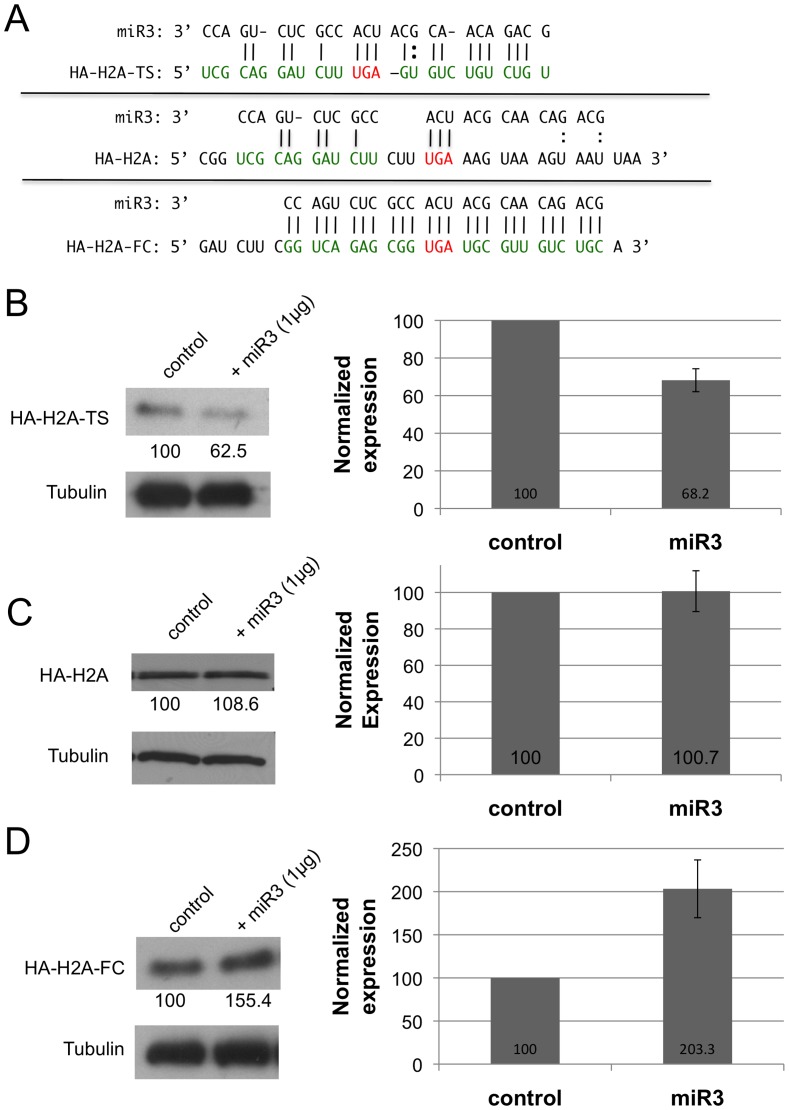
Effects of miR3 on HA-tagged histone H2A expression in *Giardia* carrying varied target sites. **A)** A diagram of the three different target sites carried by the HA-H2A transcript showing their base-pairings with miR3. **B, C and D)** The effects of miR3 on the expression of HA-H2A carrying different target sites were monitored by Western blot (left panel) with the mean intensity +/− SD of individual stained bands from three independent experiments analyzed by densitometry (right panel). **B)** HA-H2A-TS: **C)** HA-H2A; **D)** HA-H2A-FC. The controls in **B)**, **C)** and **D)** show the levels of expression of different transcripts without co-introduction with miR3. They are each set at 100%.

The enhanced expression of a reporter transcript by miR3 prompted us to look at the potential effect of miR3 on the expression of an endogenous transcript carrying a fully complementary target in Giardia. We first episomally expressed an N-terminally HA-tagged histone H2A gene carrying its original target site (HA-H2A-TS) under the control of a tetracycline inducible promoter [30]. Two additional mutants were added to the study. One has a portion of the original target site downstream from the stop codon deleted so that the seed sequence of miR3 cannot bind to the truncated target site (HA-H2A). The other has the target site converted to a sequence fully complementary to that of miR3 (HA-H2A-FC) (Figure 2A). Expression of the three constructs in Giardia, monitored by Western blot and immunofluorescence microscopy, showed that HA-H2A was expressed by all three constructs (data not shown).

When synthetic miR3 was introduced into the transfectants, expression of HA-H2A-TS was repressed by 31.8% (Figure 2B), HA-H2A remained relatively unchanged at 100.7% (Figure 2C), whereas HA-H2A-FC was enhanced to 203.3% of the original level (Figure 2D). These results are in good agreement with those from the previously observed RLuc expression (Figure 1). Thus, binding of miR3 to a fully complementary target site at the 3′ end of H2A mRNA also leads to a 2-fold enhanced expression.

#### The effects of other miRNAs in *Giardia* on their respective fully complementary targets

In an effort to understand the mechanism of miR3-mediated activation, we initially looked at the estimated free energy (ΔG) of miR3 binding to its fully complementary target site, which is −52.43 kcal/mole. This is very similar to that of miR2 binding (ΔG = −52.22 kcal/mole), miR4 binding (ΔG = −50.29 kcal/mole) [24], miR5 binding (ΔG = −52.42 kcal/mole) [22], miR6 binding (ΔG = −52.78 kcal/mole) and miR10 binding (ΔG = −56.14 kcal/mole) to their respective fully complementary targets (Figure S2A). Bindings of miR4 [24] and miR5 [22] to their fully complementary targets have been shown to repress translation to a similar extent as binding to the native target sites [22], [24]. The effects of miR2, miR6 and miR10 on their respective fully complementary targets were tested in the current study. Both miR6 and miR10 repressed the expression of target-site carrying RLuc mRNA by ∼20% (Figure S2B). But miR2 enhanced the expression by ∼30% (Figure S2B). Quantitative RT-PCR (RT-qPCR) analysis of the reporter mRNAs indicated no significant change in the mRNA levels with the introduction of miRNAs, suggesting translational repression by miR6 and miR10 but activation by miR2 (Figure S2C). There are thus two miRNAs in *Giardia*, miR3 and miR2, showing activation of translation of an mRNA carrying fully complementary target sites.

### Mutations of the Fully Complementary Target of miR3

**Figure 3:**
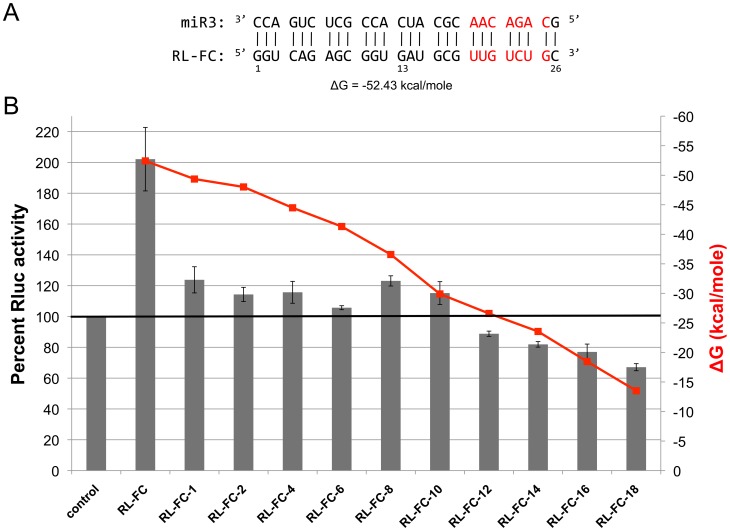
Analysis of mutations to a fully complementary (FC) miR3 target. **A)** The numberings of nucleotides in FC with the seed sequence shown in red. The ΔG derived from base-pairings with miR3 is shown below. **B)** Mutations of RL-FC were carried out stepwise from the 5′-end of FC to reduce the base-pairings with miR3. Mutant transcripts were transfected into *Giardia* trophozoites along with miR3 and assayed for RLuc activity. The mean +/− SD from three independent experiments is presented. The ΔG values estimated from base-pairings with miR3 for each mutant is shown in an orange-colored line. The control column on the left represents the level of expression of each of the mutant transcripts in the absence of introduced miR3. They are each set at 100% and presented in a single column.

Successive alterations of the fully complementary target of miR3 were carried out by mutations starting from the 5′-end of the target (Figure 3A). A disruption of the very first base-pair (RL-FC-1) (Figure S3) reduced the miR3-mediated activation by 76.2% (Figure 3B). Further disruption of the base-pairs in 2 nt increments up to the first 10 nts from the 5′-end of the target site (Figure S3), however, showed only slight variations in the reduced activation. This reduction ranged from between 76.9% (RL-FC-8) to 94.3% (RL-FC-6) and is within experimental error (Figure 3B). Thus, full complementation between miR3 and the target throughout the entire 26 base-pairs (bp) is apparently essential for the full 2-fold activation, whereas a residual ∼20% activation is maintained when complementation is disrupted along the first 10 bp. A steady linear reduction of the free energy of binding was calculated from −49.36 to −29.90 kcal/mole upon the stepwise loss of complementation along the 10 bp (Figure 3B), suggesting that free energy of binding is not related to the activating effect of miR3.

Further disruption of base-pairings from the 12^th^ to the 18^th^ nt from the 5′-end of the target sequence (Figure S3) showed repression of RLuc expression increasing from 11.2% (RL-FC-12) to 36.9% (RL-FC-18) (Figure 3B). This is correlated with the estimated ΔG of miR3-target binding from −26.60 to −13.51 kcal/mole, suggesting that higher free energy of binding leads to more efficient repression. This interesting finding agrees with our previous finding that miR3 exerted little effect on the expression of potassium transporting ATPase alpha chain 1 transcript (see Figure 1A), which has a target site fully complementary to the miR3 from nt 14 to 25 (Table 1).

To verify whether the miR3-mediated repression of RL–FC-18 expression requires GlAgo, the experiment was repeated in the GlAgo KD cells as previously described (see Figure 2B). No apparent effect of miR3 was observed in these cells (data not shown), indicating an essential role of GlAgo.

Further loss of complementation with the miR3 seed sequence in the target abolished all the effect of miR3 on RLuc expression (data not shown), suggesting that full base-pairings between nts 19 and 26 in the target and nts 1 and 8 in miR3 are essential for the repressive function of miR3.

### The Seed Sequence of miR3 Alone is Sufficient to Repress Translation

**Figure 4:**
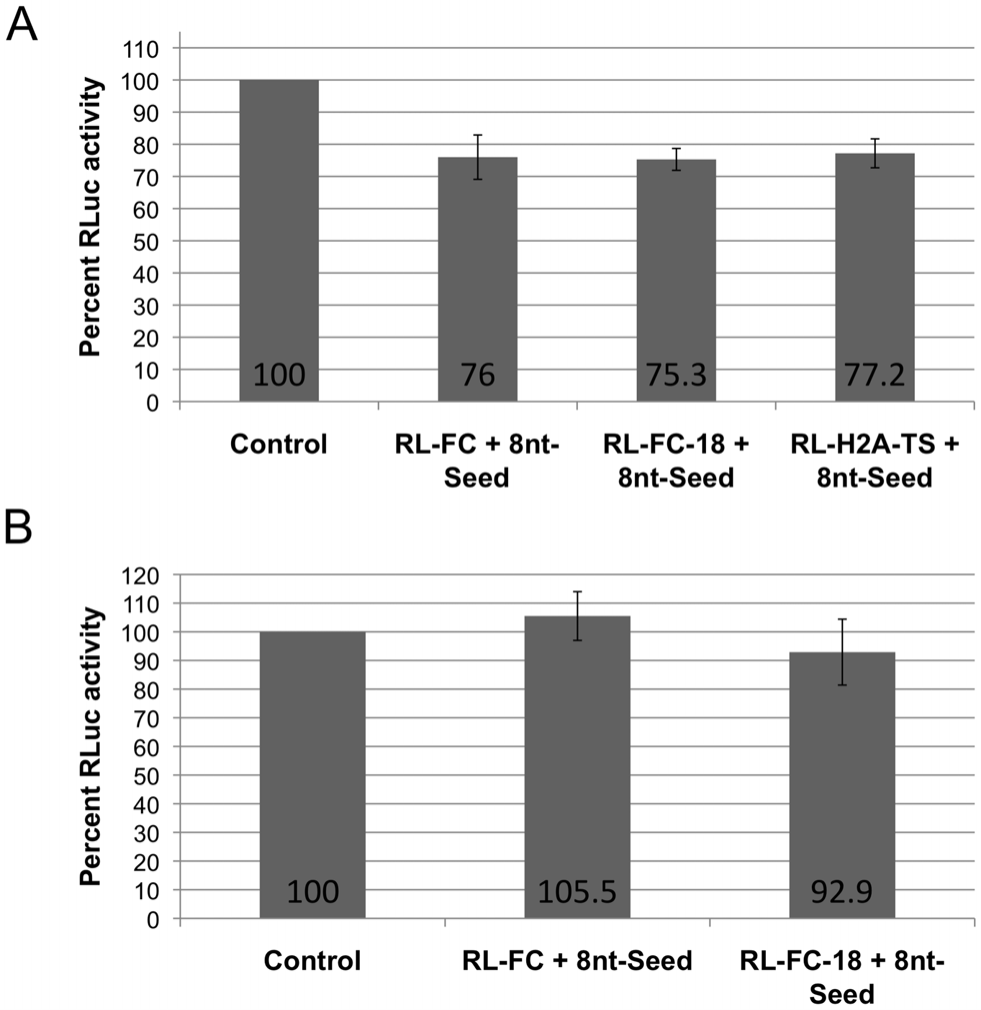
Repression of RLuc transcript expression by the miR3 seed oligomer (8 nt-Seed). **A)** An 8 nt miR3 seed oligomer (8 nt-Seed) is capable of repressing expression of RL-FC by 24%, RL-FC-18 by 24.7% and RL-H2A-TS by 22.8%. **B)** The 8 nt-Seed was also tested on RL-FC and RL-FC-18 expression in the GlAgo KD cells, and showed no apparent effect. The data represent the mean +/− SD from at least two independent assays. The controls show the levels of expression of various chimeric transcripts in the absence of exogenously introduced 8nt-Seed. The values are set at 100% and presented in a single control column.

The maximum repressive effect of miR3 demonstrated on the expression of RL-FC-18 (Figure 3B) indicates that the mere formation of a seed duplex (nt 1 to 8) could be sufficient for maximum translation repression. We chemically synthesized an 8-nt oligomer corresponding to the seed sequence (nt 1–8) of miR3 (8-nt Seed). This 8-nt Seed was tested on *Giardia* cells expressing RL-FC or RL-FC-18 and found to inhibit the RLuc expression by ∼25% (Figure 4A). When the experiment was repeated in GlAgo knockdown cells, the repressive effect was largely abolished (Figure 4B), suggesting the dependence of 8-nt Seed action on GlAgo. When this oligomer was tested on the expression of RL-H2A-TS (see Figure 1A), it showed a ∼23% repression (Figure 4A) similar to that observed on RL-FC and RL-FC-18. Thus, an 8-nt Seed oligomer works well on targets of widely varying upstream sequences as long as formation of the seed duplex remains unaffected.

Thus, in essence, we have made an interesting observation that miR3 requires only its seed sequence to form a duplex with the target to exert translational repression. The seed sequence itself is capable of performing the repressive function of miR3 without the rest of the 3′-sequence. The function of this 8-nt Seed oligomer, also mediated by Argonaute, may provide a new approach for us to look at the mechanism of miRNA repression. More intriguingly, we also observed that when miR3 binds to a fully complementary target, it exerts a strong GlAgo-dependent activation of translation instead of a reduction. This is the first time as far as we are aware that a miRNA could exert totally opposite regulatory effects on transcript translation depending solely on the extent of its interaction with the target.

## Discussion

Here we present the first evidence of a biphasic miRNA that can either repress or activate translation based solely on the extent of its base-pairings with the target. Both actions are dependent on the presence of an Argonaute protein, GlAgo, suggesting formation of a functional RNA-induced silencing complex (RISC) including the miRNA, the target and GlAgo for both actions.

### The Repressive Function of miR3

The miR3 mediated translational repression of histone H2A in Giardia raises the question on potential global effect of miR3 on chromosomal remodeling. Basic chromatin fibers were found in typical nucleosomal conformations in Giardia trophozoites [31]. They consist of four core histones H1, H2B, H2A and H4 [31]. But extensive chromosome rearrangements [32] and genome plasticity among many of the chromosomes [33] have made investigation of chromosomal remodeling difficult at the present time.

A maximum 36.9% repression of translation is achieved when only the 8 nt seed sequence in miR3 hybridizes to the target (RL-FC-18). It strongly suggests that the sequence downstream from the seed region in miR3 is not required for its repressive function. When an 8-nt oligomer corresponding to the miR3 seed sequence was introduced into *Giardia* cells, a similar GlAgo-dependent repression of ∼25% on the expression of RL-FC-18 or RL-H2A-TS was observed (Figure 4). These observations raise an interesting possibility that only the seed sequence of a miRNA and the Argonaute protein is required to form a complex with the target to repress translation of a transcript in *Giardia* and that an 8-nt oligomer is sufficient to interact with GlAgo.

Previous studies have shown that a 9 nt guide DNA complexed with the *Thermus thermophilus* Argonaute protein was sufficient to promote target RNA cleavage *in vitro* [34]. A 2.6 Å crystal structure of the PAZ domain from human Argonaute (hAgo) complexed with a 9 bp RNA duplex with a 3′-2 nt overhang in the DNA guide strand was recently analyzed [35]. These data indicate that a small RNA fragment is capable of binding to the Argonaute and forming a duplex with the target. But there has not yet been any previous indication, to our knowledge, that the seed fragment of a miRNA is capable of performing the full function of a miRNA in cells. The current observation could be the first of such an example. It could be a specific phenomenon confined to the ancient protozoan *Giardia*, which may cast some interest in the evolutionary aspect of miRNA function. Or, if it turns out to be true in other organisms as well, it may shine new lights on our current thinking of the mechanism of miRNA action.

### The Activating Function of miR3

There have been many examples showing that a small RNA could function as either a small interfering (si)RNA or a miRNA depending on the extent of base-pairings with the target [36]. A single splicing Argonaute protein could thus fulfill the role in an RISC for both siRNA and miRNA actions. Crystallographic data shows that the presence of a mismatch at positions 10 or 11 in the miRNA-target duplex places a bulge near the catalytic DDE triad in the PIWI domain of *T. thermophilus* Argonaute protein, resulting in resistance of the target RNA to Argonaute slicing [34]. The existence of non-slicing Argonautes has been nevertheless demonstrated. In humans, only hAgo2 is capable of slicing mRNA [37], [38], even though the conserved DDE catalytic triad is present in hAgo1, hAgo2 as well as hAgo3 [37]. In a recent report, the crystal structure of *Kluyveromyces polysporus* Argonaute was shown to possess a hydrogen-bond network that stabilizes an expanded and repositioned loop L2, inserting a glutamate into the catalytic pocket [39]. This insertion of a glutamate finger apparently completes a catalytic tetrad, activating the Argonaute protein for RNA cleavage [39]. This particular structural feature could distinguish the slicing from the non-slicing type of Argonaute.

GlAgo appears to possess the DDE triad, but has not shown any mRNA slicing activity suggesting that GlAgo may be missing the structure to form the catalytic tetrad for RNA slicing [22], [24], [39]. Using mRNAs containing fully complementary target sites, none of the corresponding miRNAs demonstrated any RNAi function (Figure S2B). miR4, miR5, miR6 and miR10 repressed translation to similar extents as those with the imperfect native target sites, whereas miR2 and miR3 stimulated translation when the target sites were fully complementary. The basis behind these opposite actions of miRNAs in *Giardia* remains unclear. A human miRNA miR-326 has recently been found to act on the predicted target site in HIV genome and decrease the viral replication [40]. When the degree of complementarity between the miRNA and the viral target increases, the extent of repression of HIV replication also increases, thus providing an example contrary to what we have observed.

Examples of miRNA-enhanced gene expression have been recently observed in other organisms. An increasing number of studies have indicated that miRNAs can stimulate posttranscriptional gene expression regulated by the RNA sequence associated with micro-ribonucleoproteins [41]. Notably, human miR369-3 was found to activate translation of TNF-α mRNA during cell cycle arrest, but repress the same translation in proliferating cells without affecting the mRNA levels [42], [43]. The target site is near an AU-rich element and does not form perfect base pairings with miR369-6. The oscillating functions of this miRNA could be attributed to the changing availability of factors required for the opposing effects in different phases of cell development. The liver-specific miR122 binds to two consecutive target sites in the 5′-UTR of hepatitis C virus RNA and stimulates expression of the viral RNA without affecting its level [44], [45]. Only the seed region and nts 14–16 in the miRNA are required for base-pairings with the target for the activating function [44]. The reason why target sites located at the 5′-UTR of hepatitis C virus transcript can mediate the stimulatory function of a miRNA is attributed to the presence of an internal ribosome entry site instead of a cap at the 5′-UTR of the viral transcript. Thus, though the more detailed mechanisms of action are still unclear, it is apparent that these two examples of translation activation by miRNAs are associated with specific temporal or spatial circumstances and not dependent on full base pairings with the target.

An Argonaute protein is composed of an amino-terminal N and PAZ domains and carboxy-terminal Mid and PIWI domains. The crystal structure of an Argonaute Asn546 catalytically inactive mutant protein from *T. Thermophilus* was recently analyzed [46]. Since the mutant protein has lost its slicing activity, a stable ternary complex of the protein with a 5′-phosphorylated 21 nt guide DNA and a perfectly complementary 19 nt target RNA was formed and examined at 2.8Å resolution. The guide-target duplex starting from the seed region and spanning from position 2 to 16 in a Watson-Crick A-form helical structure was anchored in the Mid pocket with the scissile phosphate between nts 10 and 11 in the target strand positioned opposite to the catalytic residues DDE in the PIWI domain as anticipated. The N domain, however, blocked propagation of the duplex structure beyond bp16, causing apparent separation between the guide and target strands at this point. The maximum length of the guide-target duplex in the *T. thermophilus* Argonaute crystal structure is thus limited to 16 bp. Eukaryotic Argonautes are capable of binding to longer RNA duplexes. Recently, the full-length crystal structure of hAgo2 was resolved [47]. It positions the N domain away from the central grove and thus allows for a more extended RNA duplex to bind [47]. A similar observation of *K. polysporus* Ago also indicated a lengthening and slight widening of the duplex-binding channel by pushing the N domain aside to accommodate a longer RNA duplex [39].

To gain a better understanding of the potential interaction between a miR3 duplex and GlAgo, we used homology modeling to predict a potential structure of GlAgo. The relatively low sequence homology between GlAgo and the other available Argonaute crystal structures led us to use three different modeling servers; the I-TASSER homology-modeling server [48], the Phyre2 and the ModWeb servers [49], [50]. The three predicted structures of GlAgo were all based on the *T. thermophilus* Ago crystal structure and appear similar (Figure S4A), providing some confidence in this predicted structure. The predicted structure also agrees well with the hAgo2 crystal structure [47] with the N, PAZ, Mid and PIWI domains structurally well conserved (Figure S4A). The Mid and PIWI domains showed the most structural conservation among the models while the N and PAZ domains are less conserved probably due to the lower sequence homology (Figure S4A).

The I-TASSER model of GlAgo overlaps well with the crystal structure of *T. thermophilus* Ago (Figure S4B) [46], [48]. A 26 bp RNA duplex was modeled into the GlAgo structure using the crystal structure of *T. thermophilus* Argonaute Asn546 mutant guide-target duplex as a guide [46]. The duplex structure is anchored in the Mid pocket and flanked by the PAZ and PIWI domains of GlAgo as in the *T. Thermophilus* Ago (Figure S4C). The N domain in GlAgo is connected to the PAZ domain by a single loop, suggesting that the N region in GlAgo is mobile. A presumed movement of the N domain away from the central grove like that in hAgo2 and *K. polysporus* Ago would enable GlAgo to accommodate the 26 bp duplex (Figure S4C). This modeling could explain how the miRNAs, which have a relatively large size of 26–28 nts and form fully complementary 26–28 nt miRNA-target duplexes can be accommodated into GlAgo. We thus postulate that, in *Giardia*, the fully complementary 26 bp miR3-target duplex (and probably to a lesser extent the miR2 duplex) may differ from the other miRNA duplexes in interacting with the mobile N domain and induce specific structural changes in GlAgo. These changes may allow the binding of additional specific proteins to the RISC complex leading to translational activation. Other partial or mismatched miRNA-target duplexes may lead to different conformational changes of GlAgo, resulting in a protein complex for translational repression. A close examination of the sequences of these miRNA duplexes listed in Figure S2A, however, did not immediately explain how the duplexes of miR2 and miR3 may differ structurally from the rest. The detailed mechanism of miR3-mediated activation of translation remains thus unexplained at this time.

## Materials and Methods

### Incorporation of miR3 Target Sites into the RLuc Reporter Gene

Incorporation of different target sites or target site mutants was done by PCR amplification of the RLuc reporter gene. Two copies of each mutated target site were incorporated into a reverse primer (see Table S2). This reverse primer along with a forward primer containing a T7 promoter (T7 promoter Rluc) was used to PCR-amplify the RLuc reporter gene containing a target site in the 3′ UTR. The PCR product was gel purified and used for a second round of PCR to add a poly (A) tail using the T7 promoter Rluc forward primer and the miR3 comp add polyA R reverse primer. The resulting PCR product was phenol/chloroform extracted, ethanol precipitated, suspended in sterile water and then sequenced. The purified PCR product was used for capped *in vitro* transcription using the mMessage mMachine kit (Ambion) following the manufacturer directions. The transcribed RNA was precipitated with LiCl, resuspended in RNase-free water, and quantitated using a Nanodrop 2000 (Thermo Scientific).

### miRNA Assay

The assay for miRNA repression of RLuc transcript expression was performed as previously described using either *Giardia* WB wild-type or the GlAgo KD WB trophozoites [25]. *Giardia* WB trophozoites, grown in modified TYI-S-33 media to a density of 10^7^ per ml, were pelleted. Cells were washed once in phosphate buffered saline (PBS), once in electroporation buffer (10 mM K2HPO4–KH2PO4 (pH 7.6), 25 mM HEPES (free acid), 120 mM KCl, 0.15 mM CaCl2, 2 mM EGTA, 5 mM MgCl2, 2 mM ATP, 4 mM Glutathione), and finally suspended in electroporation buffer. Reporter mRNA (∼3 µg), yeast tRNA (125 µg), and, if needed, 1 µg of 5′-phosphate-miR3 RNA (miR3) (IDT) were added to the cell suspension (∼10^6^ cells), incubated on ice for 10 min and then subjected to electroporation using a Bio-Rad Gene Pulser Xcell (Voltage: 450 V, Capacitance: 500 µF, Resistance: ∞). Cells were then incubated on ice for 10 min and added to pre-warmed culture medium. The transfected cells were incubated at 37°C for 5 hours, pelleted, washed once in PBS, and lysed using the *Renilla* luciferase assay kit (Promega). The lysate was centrifuged at 13,000 g for 2 min to remove cellular debris. The cleared lysate was used to test for *Renilla* luciferase activity. The protein concentration of the cleared lysate was measured using the Bradford assay (Bio-Rad) and used to normalize the total protein concentration.

### HA-H2A-TS Cloning and Expression

The HA-tagged histone H2A gene with its putative target site (TS) for miR3 and the mutant constructs were PCR amplified from *Giardia* genomic DNA using the HA-H2A F primer and the appropriate reverse primer (see Table S2). The product was cloned into pJet2.1 (Fermentas), sequenced and sub-cloned into the pNlop4 vector, a derivative of the pNLop3-GtetR vector, using *Nco*I and *EcoR*I [30]. The resulting construct was transfected into *Giardia* trophozoites as previously described and the transfectants were selected with 200 µg/ml G418 [51]. The transfected cells were induced with 10 µg/ml of tetracycline at 37°C for 24 hours and assayed by Western blot to confirm expression of the protein.

## Supporting Information

Figure S1 **Characterization of miR3.** **A)** The FISH assay using the 5′-end 26 nt and 3′-end 26 nt sequences of the snoRNA GlsR16 as probes [25] indicates that the snoRNA is predominately localized to the nucleolus with some presence in the nucleus. miR3 is primarily localized to the cytoplasm of *Giardia* trophozoites. **B)** qRT-PCR indicates that miR3 is enriched in the small RNA band co-immunoprecipitated with HA-GlAgo [24].
(TIF)

Figure S2 Effects of all six identified *Giardia* miRNAs on translation of RLuc mRNAs carrying fully complementary target sites. A) Alignment of the six miRNAs with their respective fully complementary targets. B) Effects of the six miRNAs on expression of RLuc carrying their respective fully complementary target sites. C) qRT-PCR estimation of the levels of RLuc mRNAs following the actions of miRNAs. None of the mRNAs showed signs of reduced level, suggesting that *Giardia* miRNAs act on translation of the mRNAs.
(TIF)

Figure S3 **Sequences of different FC target mutants used in this study.** Red letters indicate the altered nucleotides and green letters specify the seed sequence in the target.
(TIF)

Figure S4 **Homology modeling of GlAgo.** **A)** The ModWeb, Phyre2 and I-TASSER models of GlAgo based on the crystal structure of T. thermophilus Ago are presented [48], [49], [50]. An I-TASSER model of GlAgo based on the crystal structure of hAgo2 is also included [47], [48]. All four models bear excellent similarities. The model analysis and image presentation was performed with UCSF Chimera [52]. **B)** The I-TASSER model of GlAgo overlapped with the crystal structure of T. thermophilus Ago (3HK2) [48]. **C)** Modeling of a 26 bp RNA duplex into the I-TASSER model of GlAgo required a slight movement of the N domain toward the right to accommodate the inserted duplex. Movement of the N domain and modeling of the 26 nt RNA duplex were performed with UCSF Chimera [52].
(TIF)

Table S1 **List of miRanda identified putative miR3 target sites in** ***Giardia*** **.**
(XLSX)

Table S2 **List of PCR primers.**
(XLSX)
