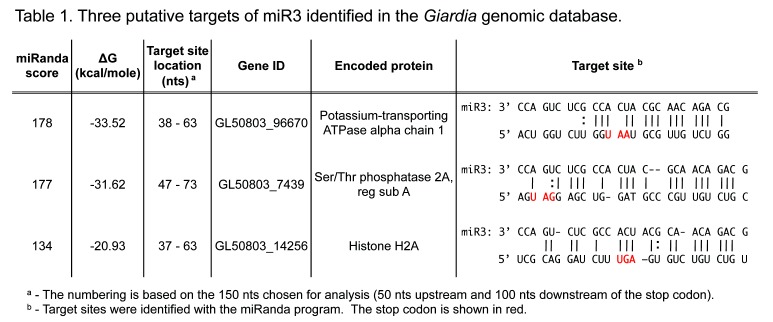# Correction: Transition of a microRNA from Repressing to Activating Translation Depending on the Extent of Base Pairing with the Target

**DOI:** 10.1371/annotation/cb23f7bd-0d8c-4fa2-8ce8-1d641c03f561

**Published:** 2013-10-24

**Authors:** Ashesh A. Saraiya, Wei Li, Ching C. Wang

Table 1 was formatted incorrectly. The correct version of Table 1 can be viewed here: 

**Figure:**