# Supplementary figures and images for: Transition of a microRNA from Repressing to Activating Translation Depending on the Extent of Base Pairing with the Target
Source: PLoS One. 2013 Feb 6;8(2):e55672. doi: 10.1371/journal.pone.0055672 (PMC3565978; doi:10.1371/journal.pone.0055672)

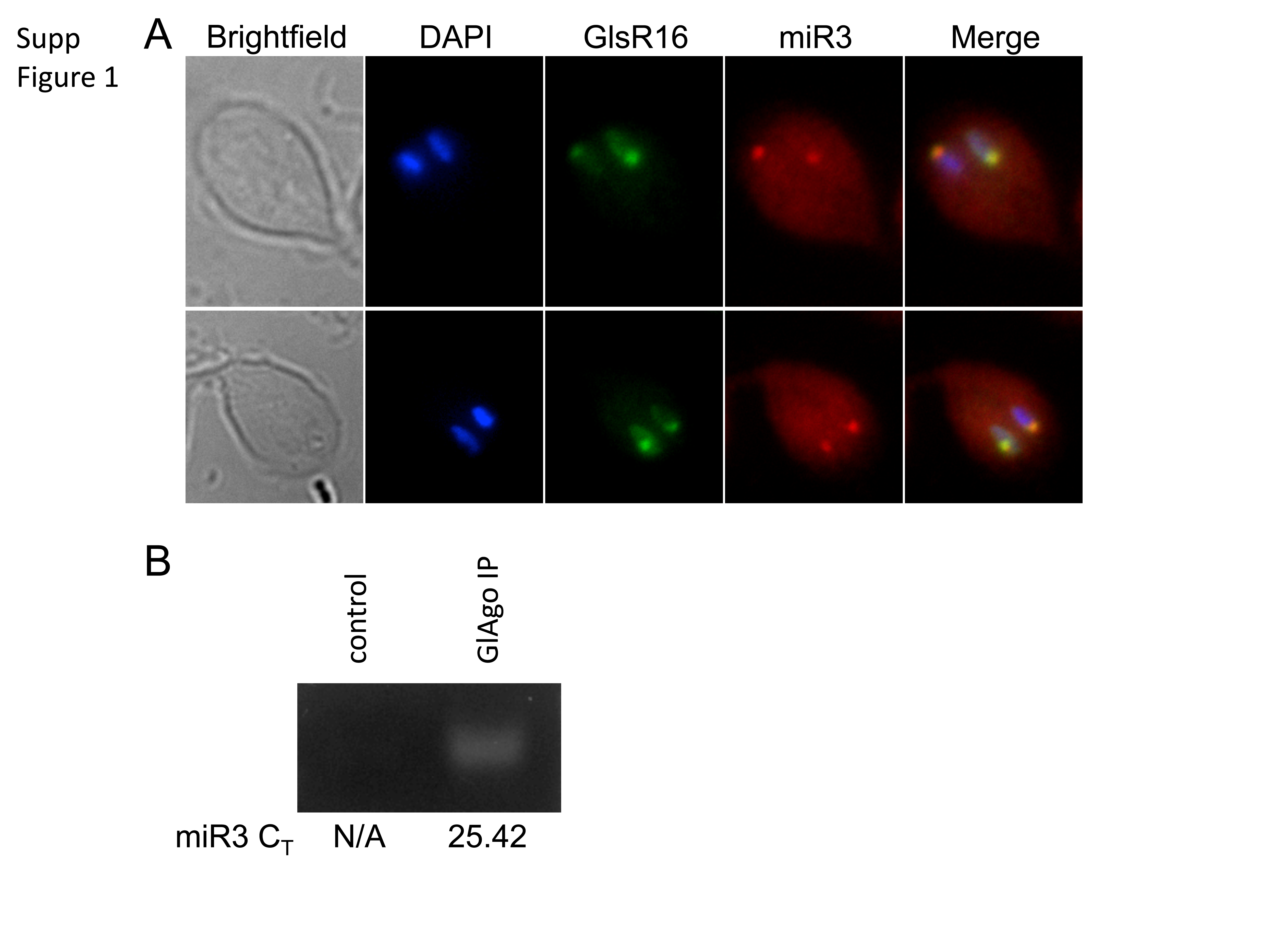

Supplement: Figure S1 — Characterization of miR3. A) The FISH assay using the 5′-end 26 nt and 3′-end 26 nt sequences of the snoRNA GlsR16 as probes [25] indicates that the snoRNA is predominately localized to the nucleolus with some presence in the nucleus. miR3 is primarily localized to the cytoplasm of Giardia trophozoites. B) qRT-PCR indicates that miR3 is enriched in the small RNA band co-immunoprecipitated with HA-GlAgo [24]. (TIF) [file pone.0055672.s001.tif]

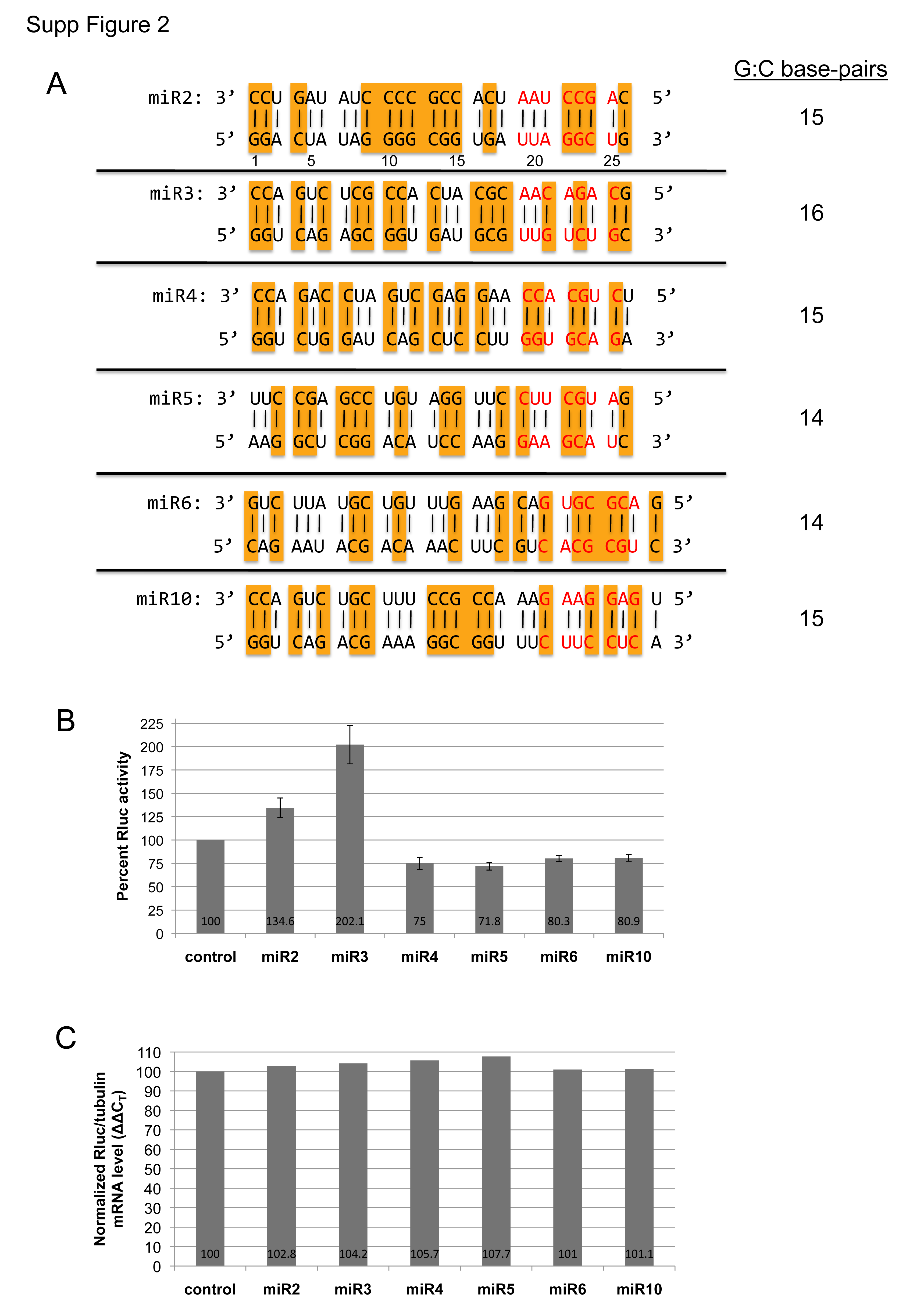

Supplement: Figure S2 — Effects of all six identified Giardia miRNAs on translation of RLuc mRNAs carrying fully complementary target sites. A) Alignment of the six miRNAs with their respective fully complementary targets. B) Effects of the six miRNAs on expression of RLuc carrying their respective fully complementary target sites. C) qRT-PCR estimation of the levels of RLuc mRNAs following the actions of miRNAs. None of the mRNAs showed signs of reduced level, suggesting that Giardia miRNAs act on translation of the mRNAs. (TIF) [file pone.0055672.s002.tif]

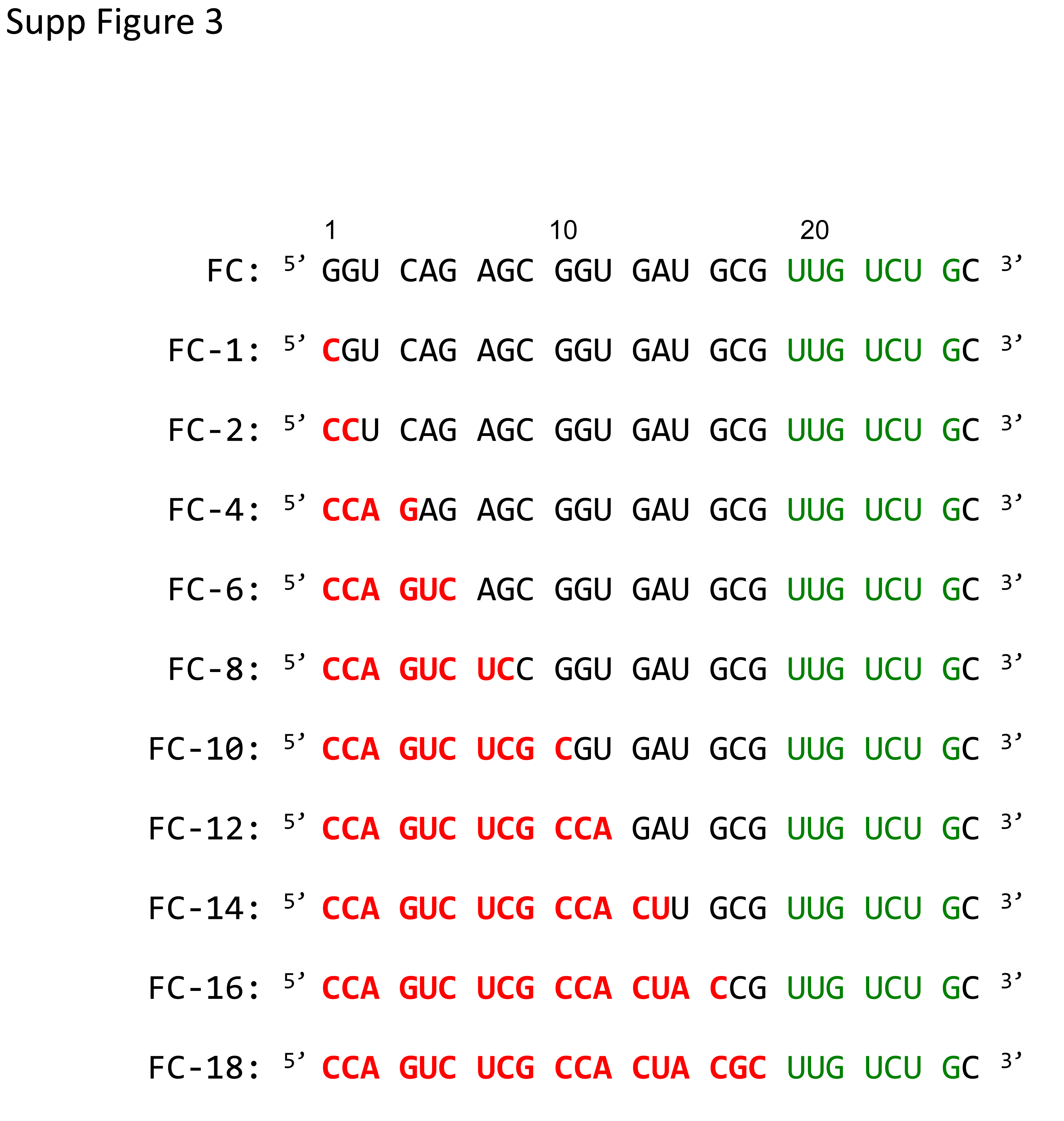

Supplement: Figure S3 — Sequences of different FC target mutants used in this study. Red letters indicate the altered nucleotides and green letters specify the seed sequence in the target. (TIF) [file pone.0055672.s003.tif]

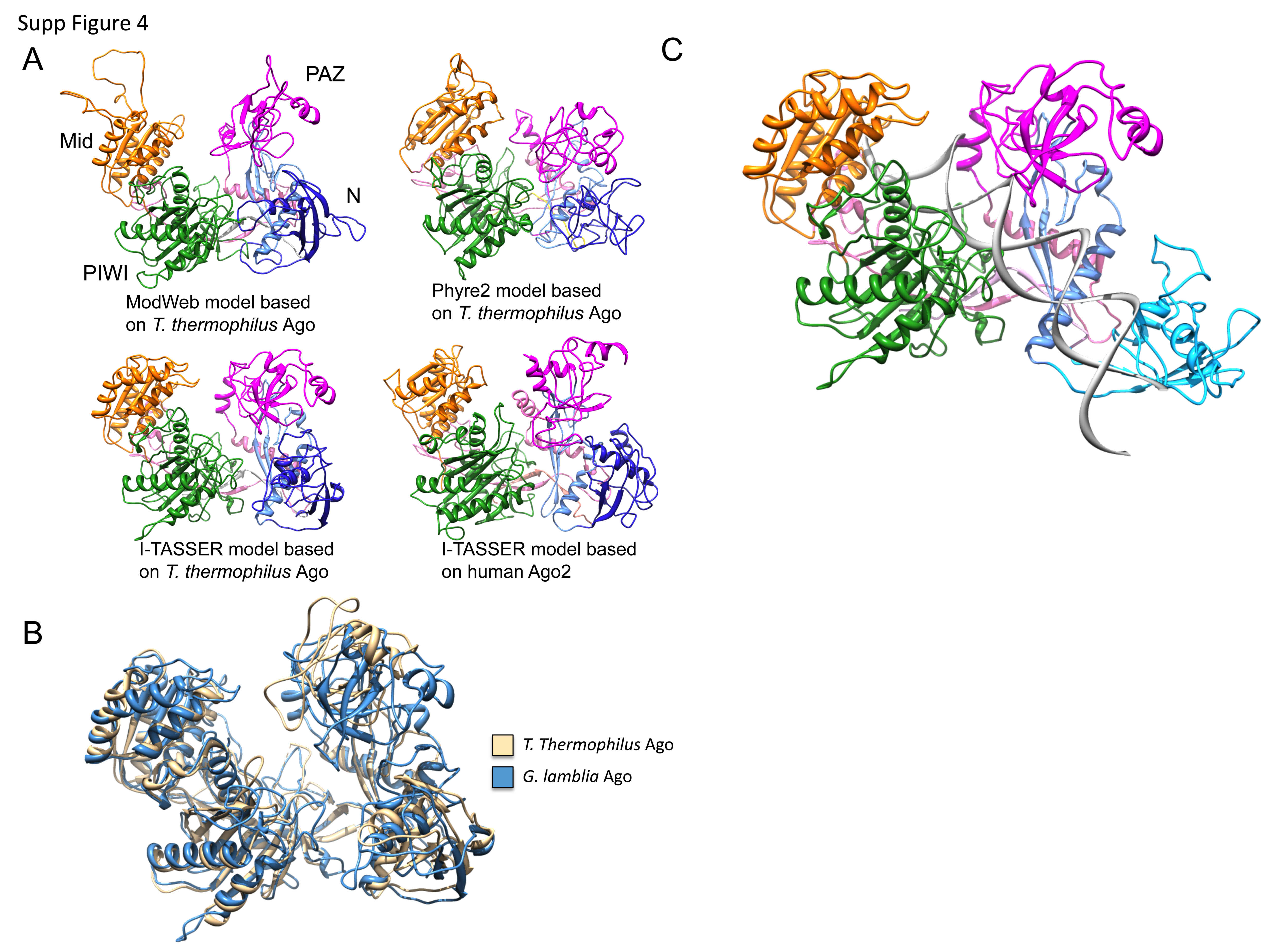

Supplement: Figure S4 — Homology modeling of GlAgo. A) The ModWeb, Phyre2 and I-TASSER models of GlAgo based on the crystal structure of T. thermophilus Ago are presented [48], [49], [50]. An I-TASSER model of GlAgo based on the crystal structure of hAgo2 is also included [47], [48]. All four models bear excellent similarities. The model analysis and image presentation was performed with UCSF Chimera [52]. B) The I-TASSER model of GlAgo overlapped with the crystal structure of T. thermophilus Ago (3HK2) [48]. C) Modeling of a 26 bp RNA duplex into the I-TASSER model of GlAgo required a slight movement of the N domain toward the right to accommodate the inserted duplex. Movement of the N domain and modeling of the 26 nt RNA duplex were performed with UCSF Chimera [52]. (TIF) [file pone.0055672.s004.tif]
